# Anatolicin, a Highly Potent and Selective Cytotoxic Sesquiterpene Coumarin from the Root Extract of *Heptaptera anatolica*

**DOI:** 10.3390/molecules24061153

**Published:** 2019-03-23

**Authors:** Fatma Tosun, John A. Beutler, Tanya T. Ransom, Mahmut Miski

**Affiliations:** 1 Department of Pharmacognosy, School of Pharmacy, Istanbul Medipol University, Istanbul 34810, Turkey; 2 Molecular Targets Program, CCR, NCI, Frederick, MD 21702, USA; beutlerj@mail.nih.gov (J.A.B.); ttrj2006@verizon.net (T.T.R.); 3 Department of Pharmacognosy, Faculty of Pharmacy, Istanbul University, Istanbul 34116, Turkey

**Keywords:** *Heptaptera anatolica*, Apiaceae, sesquiterpene coumarins, cytotoxic activity

## Abstract

Seven known sesquiterpene coumarins and a new sesquiterpene coumarin, anatolicin (**8**), were isolated from the dichloromethane extract of the roots of *Heptaptera anatolica*. Structures of these compounds were elucidated based on their spectral properties. While some of these sesquiterpene coumarins showed modest cytotoxic activity against COLO205, KM12, A498, UO31, and TC32 cancer cell lines, selective cytotoxicity of anatolicin (**8**) and 14′-acetoxybadrakemin (**7**) were observed at nanomolar level against the UO31 kidney cancer cell line.

## 1. Introduction

Cancer is the second leading cause of death worldwide according to WHO statistics [1]. Cancer is a highly complex disease that evolves through the acquisition of multiple biological capabilities by normal cells to become cancer cells [2]. In order to develop new cancer medicines, it is crucial to recognize the various pathways that provide immortality to cancer cells [3]. Natural products represent a major and highly diverse source for the development of novel cancer treatments [4]. As part of our continuing studies on potential anticancer phytochemicals from Apiaceae family, we report here the cytotoxic compounds of *Heptaptera anatolica* (Boiss.) Tutin.

Oleo-gum-resins obtained from the *Ferula* (Apiaceae) species have been used for the treatment of various tumors since ancient times; Dioscorides refers to the use of such drugs in the De Materia Medica [5], and Avicenna describes the application of *Khiltit* (the oleo-gum-resin of *Ferula foetida*) in the treatment of tumors, by direct application to the tumor after cutting it open, as described in the Al-Qanun fi al-Tibb (the Canon of Medicine) [6]. The sesquiterpene ethers found in the oleo-gum-resins of *Ferula* species are mainly responsible for their cytotoxic activities, in addition, they show many biological activities such as antiviral, antibacterial, antileishmanial, anti-inflammatory and P-glycoprotein inhibitory activities [7].

*Heptaptera* Marg. & Reuter is a small genus of the Apiaceae family represented by six species [8]. The majority of *Heptaptera* species are distributed throughout the countries of the Middle East (i.e., Turkey, Iran, Iraq, and Syria), except for *Heptaptera angustifolia* (Bertol.) Tutin and *H. colladonioides* Marg. & Reuter which are endemic to Italy and Greece, respectively. Characteristic secondary metabolites of *Heptaptera* (syn. *Colladonia*) sp. are sesquiterpene ethers of umbelliferone [9,10,11], in which the sesquiterpene moiety is either an acyclic sesquiterpene as in the case of umbelliprenin (**1**), or is a bicyclic drimane derivative, e.g., badrakemin (**4**). Similar sesquiterpene coumarins are also known from *Ferula* species, however, several drimane sesquiterpene ethers isolated from *Heptaptera* are biogenetically oxygenated at the C-14′ position that is a specific chemotaxonomic character of this genus and not been found in the sesquiterpene ethers isolated from *Ferula* species. In order to evaluate the cytotoxic activities of sesquiterpene coumarin ethers where C-14′ is oxygenated and related, compounds, we decided to investigate the dichloromethane root extract of *H. anatolica*.

## 2. Results

**Figure 1:**
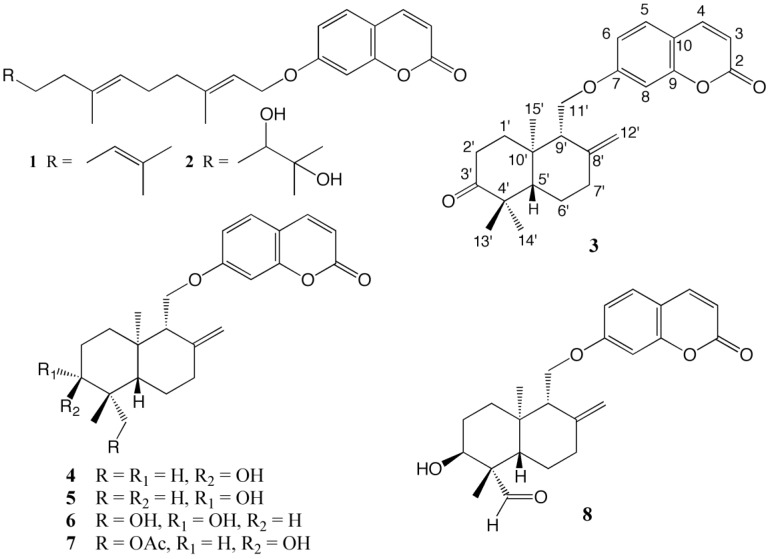
Structures of the sesquiterpene coumarins isolated from the dichloromethane extract of the roots of *Heptaptera anatolica*.

The known sesquiterpene coumarins were identified as umbelliprenin (**1**) [12], karatavicinol (**2**) [13], badrakemone (**3**) [9], badrakemin (**4**) [14], colladonin (**5**) [9], 14′-hydroxycolladonin (**6**) [9], and 14′-acetoxybadrakemin (**7**) [9] (Figure 1) by their spectral properties as well as direct comparison with the reference compounds where available. Due to their limited accessibility in the literature, high resolution ^1^H-NMR spectral data of known drimane sesquiterpene coumarins **3**–**8** are provided in Table 1 and Supplementary Materials.

## 3. Discussion

**Figure 2:**
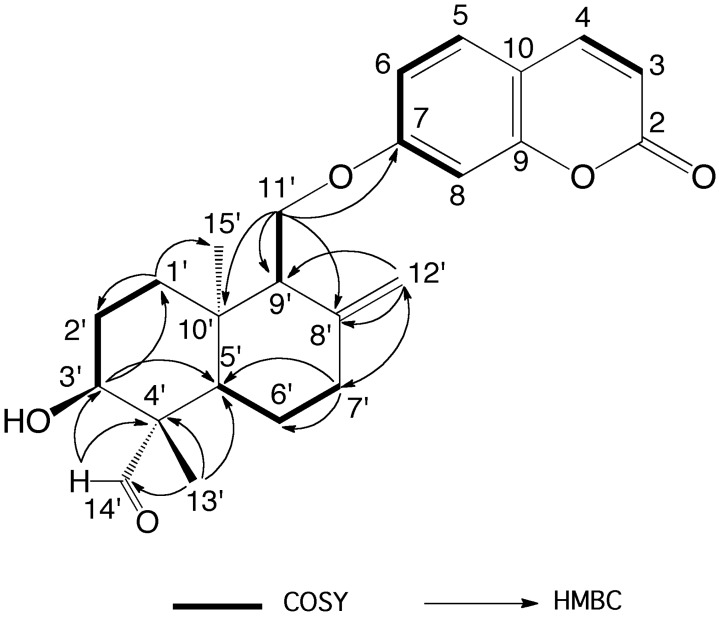
Key COSY and HMBC correlations of anatolicin (**8**).

The molecular formula of anatolicin was determined as C_24_H_28_O_5_ by the ^13^C-NMR data (Table 1) and the observed [M + H] ion at *m*/*z* 397.2010 (calcd. for C_24_H_29_O_5_, *m/z* 397.2010) indicating 11 degrees of unsaturation. The ^1^H-NMR and 2D NMR spectroscopic data of anatolicin (**8**) (Table 1, supplementary materials) were similar to that of badrakemin (**4**) (Table 2) with the exception of a missing methyl signal of the drimane sesquiterpene moiety, the presence of a tertiary aldehyde group signal at δ 9.76 ppm strongly suggesting that it was biogenetically oxidized to an aldehyde group in **8**. The strong anisotropic deshielding effect of the aldehyde carbonyl group on the chemical shifts of H-3′ and H-5′ protons (i.e., 0.70 and 0.34 ppm, respectively) in anatolicin (**8**) in comparison with those observed in badrakemin (**4**) suggests that one of the methyl groups located on the C-4′ position (i.e., C-13′ or C-14′) of the drimane skeleton must be biogenetically oxidized to an aldehyde group. The 2D HMBC spectra data (Table 1 and Figure 2) also confirm this observation.

**Figure 3:**
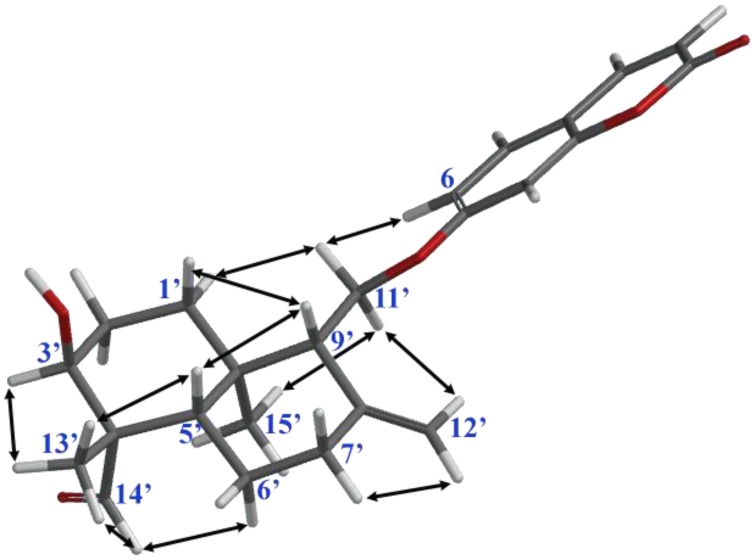
NOE interactions observed in the 2D NOESY spectrum of anatolicin (**8**) [15].

Clear interactions observed between the aldehyde proton and C-13′ and H-6α proton (Figure 3) in the 2D NOESY spectrum of anatolicin indicate that the C-14′ axillary methyl group on the C-4′ of the drimane skeleton was biogenetically oxidized to an aldehyde carbonyl, thus, anatolicin is an oxidized C-14′ derivative of badrakemin (**4**). The C-14′ oxidation pattern observed in **6**, **7**, and **8** is in agreement with the biogenetic oxidation pattern of the sesquiterpene coumarin ethers isolated from *Heptaptera* species. Although C-14′ hydroxymethylene derivatives of drimane sesquiterpene coumarin ethers have been reported previously [9,10,11], anatolicin (**8**) is the first drimane sesquiterpene coumarin ether with an aldehydic functional group.

The sesquiterpene coumarin ethers isolated from *Heptaptera anatolica* were tested against COLO205, KM12, A498, UO31, and TC32 cancer cell lines, and the cytotoxic activities observed with these compounds are shown in Table 3.

While most of the sesquiterpene coumarin ethers were active against the UO31 kidney cancer cell line, 14′-acetoxybadrakemin (**7**) and anatolicin (**8**) were the most active compounds with 17 and 24 nM IC_50_ values, respectively. Colladonin (**5**) was also active against the UO31 cell line with a 0.75 μM IC_50_ value and, furthermore, showed similar activity against the KM12 colon cancer cell line, was moderately active against the COLO205 and A498 cell lines, and weakly active against the TC32 cell line. In addition, badrakemin (**4**) also showed strong activity against the UO31 cell line and moderate activity against the KM12 and A498 cell lines.

## 4. Materials and Methods

### 4.1. General Experimental Procedures

Melting points were determined on a Reichert microscope equipped with a Kofler heating stage and were uncorrected (Wien, Austria). Optical rotations were measured using a Perkin-Elmer Model 241 polarimeter in a 100 × 2 mm cell (units 10^−1^ deg cm^2^ g^−1^) in dichloromethane. IR spectra (neat) were recorded on a Perkin-Elmer FT-IR Spectrometer, SPECTRUM 2000 (Waltham, MA, USA). NMR spectra of anatolicin (**8**) were acquired on a Bruker Avance III spectrometer (Billerica, MA, USA) operating at 600 MHz for ^1^H and 150 MHz for ^13^C and equipped with a 3 mm cryogenically cooled probe. ^1^H-NMR spectrum of compound **1** was acquired on a Bruker Avance III spectrometer (Rheinstetten, Germany) operating at 500 MHz and equipped with a 5 mm indirect observation probe and ^1^H-NMR spectra of compounds 2–7 were acquired on a Varian (Agilent) Mercury Spectrometer (Palo Alto, CA, USA) operating at 400 MHz and equipped with a 5 mm probe. ^1^H and ^13^C spectra were referenced to the residual deuterated solvent peaks. HRESIMS data were acquired on an Agilent 6530 Accurate Mass Q-TOF instrument (Santa Clara, CA, USA) and on a Triple TOF 5600 mass spectrometer (AB SCIEX, Framingham, MA, USA). Initial purification of the dichloromethane extract was carried out on a Sephadex LH-20 (GE Healthcare, Chicago, IL, USA) column. Further purification of column fractions was performed using silica gel F_254_ PLC plates (1 mm thickness) (Merck KGaA, Darmstadt, Germany).

### 4.2. Plant Material

The roots of *Heptaptera anatolica* were collected from the Izmir province in Western Anatolia in June 2013 and identified by Prof. A. Duran. A voucher specimen (A. Duran 9703) was deposited in the Herbarium of Selcuk University, Faculty of Sciences, Department of Biology.

### 4.3. Extraction and Isolation

Air-dried and coarsely powdered roots (100 g) of *H. anatolica* were extracted with dichloromethane at room temperature and concentrated, in vacuo, to dryness (5.15 g). The dichloromethane extract (4 g) was separated using a Sephadex LH-20 column (4 × 64 cm) packed in hexane/dichloromethane/methanol (14:9:1) followed by prep. TLC (1 mm thickness, silica gel F_254_ developed with hexane/ethyl acetate at 9:1, 7:3, or 1:1) for final purification of compounds. The known compounds isolated from the dichloromethane extract were umbelliprenin (**1**, 30 mg), karatavicinol (**2**, 18 mg), badrakemone (**3**, 14 mg), badrakemin (**4**, 309 mg), colladonin (**5**, 326 mg), 14′-hydroxycolladonin (**6**, 13 mg), and 14′-acetoxybadrakemin (**7**, 345 mg).Anatolicin (**8**, 38 mg). White needles from hexane/dichloromethane/methanol (14:9:1), m.p. 139–141 °C; ${\left[\alpha \right]}_{D}^{20}$−21.8 (c 2.25, CH_2_Cl_2_); IR (NaCl) ν_max_: 3488, 3112, 2932, 2900, 1733, 1709, 1612, 1556, 1509, 1398, 1352, 1282, 1230, 1015, 892, 835 cm^−1^; UV (MeOH) λ_max_ (log ε): 322 (4.11), 297 (sh) (3.86), 253 (sh) (3.25), 219 (sh) (4.02); ^1^H and ^13^C-NMR (see Table); HRESIMS *m*/*z* 397.2010 [M + H]^+^ (calcd. for C_24_H_28_O_5_ [M]^+^; 396.1937; err. 0.05 ppm).

### 4.4. Cytotoxic Activity Assay

The primary assay used for this study was a two-day, two-cell-line XTT bioassay [16], an in vitro antitumor colorimetric assay developed by the MTP Assay Development and Screening Section. Renal cancer cell lines used were UO31 and A498. Cells were harvested and plated (45 μL) at a seeding density of 1.5 × 10^4^ cells per well for both the UO31 and A498 cell line into a 384-well “assay plate” and then incubated for 24 h. DMSO solutions of the test materials (8 μL) were diluted 1:25 with medium (192 μL) and then subjected to five 2:1 serial dilutions (100 μL each) on a 96-well plate. Duplicate 40 μL aliquots of each sample concentration were then transferred to a 384-well “dilution plate”, which could accommodate the duplicate samples from two 96-well plates. A 5 μL aliquot of each solution on the dilution plate was transferred to the cell cultures on the 384-well assay plate to give a final volume of 50 μL and a DMSO concentration of 0.4%. Cells were incubated for 48 h at 37 °C in the presence of the test samples and then treated with the tetrazolium salt XTT (2,3-bis[2-methoxy-4-nitro-5-sulfophenyl]-2H-tetrazolium-5-carboxanilide). Viable cells reduced the XTT to a colored formazan product, and after an additional 4 h incubation period, the amount of formazan produced was quantified by absorption at 450 nm using the absorption at 650 nm as a reference. Sanguinarine was used on each plate as a positive control. Similar methodology and seeding densities were used for COLO205, KM12 (colon), A673, and TC32 (Ewing sarcoma) cell lines.

## 5. Conclusions

Investigation of the dichloromethane extract of the roots of *Heptaptera anatolica* yielded seven known and one new sesquiterpene coumarin. Although cytotoxic activity of sequiterpene coumarins were described in the literature earlier [7], high potency and selectivity of C-3′-β-hydroxy and C-14′-acetoxy or C-14′-keto derivatives (i.e., 14′-acetoxybadrakemin (**7**) and anatolicin (**8**)) toward UO31 kidney cancer cell line had not been reported previously.

## Figures and Tables

**Table 1:** ^1^H-NMR data of compounds **3**–**7** (400 MHz, δ in ppm, m, *J* in Hz).

Position	3 (in CDCl_3_)	4 (in CDCl_3_)	5 (in CDCl_3_)	6 (in CD_3_OD)	7 (in CDCl_3_)
3	6.25; d; 9.7; 1H	6.23; d; 9.5; 1H	6.24; d; 9.5; 1H	6.24; d; 9.6; 1H	6.23; d; 9.6; 1H
4	7.63; d; 9.7; 1H	7.62; d; 9.5; 1H	7.62; d; 9.5; 1H	7.88; d; 9.6; 1H	7.62; d; 9.6; 1H
5	7.36; d; 9.2; 1H	7.34; d; 9.5; 1H	7.35; d; 8.6; 1H	7.52; d; 8.6; 1H	7.34; d; 9.4; 1H
6	6.82; dd; 9.2, 2.4; 1H *	6.82; dd; 9.5, 2.4; 1H *	6.82; dd; 8.6, 2.4; 1H *	6.90; dd; 8.6, 2.4; 1H *	6.82; dd; 9.4, 2.3; 1H *
8	6.82; bd; 2.2 1H *	6.82; bd; 2.1; 1H *	6.81; bs, 1H *	6.92; bd, 2.3; 1H *	6.81; bd; 2.2 1H *
1′α	2.09; ddd; 3.6, 6.6, 13.2; 1H **	1.50; dt; 13.2, 3.3; 1H	1.45; m; 1H **	1.73; m; 1H **	1.73; m; 1H **
1′β	1.81; dt; 5.5, 13.2; 1H	1.83; bdt; 3.2, 13.2; 1H	1.76; m; 1H ***	1.87; m; 1H ***	1.84; dt; 2.8, 13.1; 1H
2′α	2.68; ddd; 6.9, 13.2, 15.1; 1H	1.67; m; 1H **	1.45; m; 1H **	1.73; m; 1H **	1.73; m; 1H **
2′β	2.41; ddd; 3.2, 5.3, 15.1; 1H	1.67; m; 1H **	1.76; m; 1H ***	1.87; m; 1H **	1.73; m; 1H **
3′	-	3.46; bt; 2.6; 1H	3.30; dd; 4.4, 11.4; 1H	3.43; bdd; 6.6, 10.0; 1H	3.79; bt; 2.2; 1H
5′	1.67; dd; 2.4, 12.1; 1H	1.66; dd; 2.4, 12.9; 1H **	1.17; dd; 2.7, 12.5; 1H	1.41; dd; 3.9, 12.6; 1H ****	1.54; dd; 3.1, 12.3; 1H
6′α	1.57; m; 1H	1.42; dq; 4.4, 14.0; 1H	1.62; m; 1H	1.41; m; 1H ****	1.42; dq; 4.3, 13.2; 1H
6′β	1.73; m; 1H	1.96; ddt; 2.4, 3.7, 14.0; 1H	1.76; m; 1H ***	1.87; m; 1H ***	1.93; ddt; 2.2, 2.8, 13.2 1H
7′α	2.53; ddd; 2.3, 4.0, 13.3; 1H	2.45; ddd; 2.5, 4.2, 13.2; 1H	2.46; ddd; 2.3, 4.0, 13.2; 1H	2.46; ddd; 2.3, 3.9, 13.0; 1H	2.46; ddd; 2.3, 4.1, 13.3; 1H
7′β	2.14; bdt; 4.8, 13.3; 1H **	2.13; bdt; 5.0, 13.2; 1H	2.09; bdt; 5.1, 13.2; 1H	2.07; bdt; 5.5, 13.2; 1H	2.15; bdt; 5.0, 13.3; 1H
9′	2.30; bt; 5.4; 1H	2.33; bdd; 4.2, 7.3; 1H	2.20; bdd; 4.3, 7.6; 1H	2.22; bdd; 3.6, 6.9; 1H	2.34; bdd; 4.5, 6.9; 1H
11′a	4.24; dd; 6.8, 9.8; 1H ***	4.23; dd; 4.3, 10.0; 1H	4.21; dd; 4.3, 9.7; 1H	4.28; dd; 3.9, 10.3; 1H	4.22; dd; 4.3, 9.9; 1H
11′b	4.17; dd; 5.2, 9.8; 1H ***	4.16; dd; 7.6, 10.0; 1H	4.17; dd; 7.6, 9.7; 1H	4.22; dd; 7.7, 10.3; 1H	4.17; dd; 7.4, 9.9; 1H **
12′a	4.97; bs; 1H	4.90; bd; 1.3; 1H	4.91; bs; 1H	4.89; bs; 1H	4.90; bs; 1H
12′b	4.60; bs; 1H	4.53; bd; 1.3; 1H	4.53; bs; 1H	4.56; bs; 1H	4.54; bs; 1H
13′	1.12; s; 3H	0.98; s; 3H	0.81; s; 3H	1.23; s; 3H	1.09; s; 3H
14′a	1.06; s; 3H	0.86; s; 3H	1.02; s; 3H	4.13; d; 11.1; 1H	4.18; d; 11.3; 1H **
14′b				3.39; d; 11.1; 1H	3.96; d; 11.3; 1H
15′	1.04; s; 3H	0.85; s; 3H	0.84; s; 3H	0.85; s; 3H	0.84; s; 3H
OAc	-	-	-	-	2.04; s; 3H

*, **, ***, **** Partially overlapped signals.

**Table 2:** ^1^H-NMR (600 MHz), ^13^C-NMR (150 MHz), and HMBC data of anatolicin (**8**) (in CDCl_3_).

Position	δ_H_ (in ppm, m, *J* in Hz)	δ_C_ (in ppm)	HMBC (H -> C) Correlations
2	-	161.41	
3	6.25; d; 9.7; 1H	113.22	C-2; C-10
4	7.63; d; 9.7; 1H	143.32	C-2; C-5; C-9; C-10
5	7.35; d; 8.3; 1H	128.90	C-4; C-7; C-9
6	6.82; dd; 8.3, 2.2; 1H *	113.19	C-7; C-8; C-10
7	-	162.21	
8	6.81; bs, 1H *	101.46	C-7; C-9; C-10
9	-	156.00	
10	-	112.64	
1′α	1.57; dt; 13.2, 3.3; 1H	31.85	C-2′; C-15′
1′β	1.83; ddd; 3.3, 13.2, 13.2; 1H		
2′α	1.73; m; 1H	26.65	C-1′
2′β	1.92; m; 1H		
3′	4.16; bt; 3.1; 1H **	69.10	C-1′; C-5′
4′	-	52.42	
5′	2.00; dd; 2.8, 13.3; 1H	48.60	C-4′; C-6′; C-13′; C-14′; C-15′
6′α	1.76; m; 1H	23.12	C-5′; C-7′
6′β	1.96; m; 1H		
7′α	2.53; ddd; 2.6, 4.5, 13.2; 1H	37.78	C-5′; C-6′; C-8′; C-9′; C-12′
7′β	2.15; ddd; 4.5, 13.2, 13.2; 1H		
8′	-	145.69	
9′	2.36; bdd; 3.5, 7; 1H	53.58	C-8′; C-10′; C-11′; C-12′
10′		39.30	
11′a	4.23; dd; 4.0, 9.8; 1H	65.67	C-7; C-8′; C-9′; C-10′
11′b	4.17; dd; 7.8, 9.8; 1H **		
12′a	4.96; bs; 1H	108.68	C-7′; C-8′; C-9′
12′b	4.58; bs; 1H		
13′	1.15; s; 3H	20.13	C-3′; C-4′; C-5′; C-14′
14′	9.76; s; 1H	204.49	C-3′; C-4′
15′	0.74; s; 3H	14.34	C-1′; C-5′; C-9′; C-10′

*, ** Partially overlapped signals.

**Table 3:** Cytotoxic activities of sesquiterpene coumarin ethers isolated from *Heptaptera anatolica.*

Compounds	Cytotoxic Activity (IC_50_ values in μM)					
	COLO205	KM12	A498	UO31	A673	TC32
**1**	>50	>50	>50	1.8	>50	>50
**2**	>50	>50	>50	7.6	>50	>50
**3**	>50	>50	>50	11	>50	>50
**4**	>50	9.1	20	0.38	>50	>50
**5**	19	2.5	21	0.75	>50	45
**6**	>50	>50	>50	>50	>50	>50
**7**	>50	>50	>50	0.017	>50	>50
**8**	>50	>50	>50	0.024	>50	>50

## Supplementary Materials

The Supplementary Materials are available online. the 1D and 2D NMR data, HRESIMS data of anatolicin (**8**), as well as the 1H-NMR spectra of compounds **1**–**7**.
