# Supplementary material for: Anatolicin, a Highly Potent and Selective Cytotoxic Sesquiterpene Coumarin from the Root Extract of Heptaptera anatolica
Source: Molecules. 2019 Mar 23;24(6):1153. doi: 10.3390/molecules24061153 (PMC6471333; doi:10.3390/molecules24061153)
Supplement: Supplementary file 1 [file molecules-24-01153-s001.pdf]

Supplementary Material

Anatolicin, a Highly Potent and Selective Cytotoxic Sesquiterpene Coumarin, from the Root Extract of *Heptaptera anatolica*

Fatma Tosun[1], John A. Beutler[2], Tanya Ransom[2], and Mahmut Miski[3]

Affiliation

[1]Istanbul Medipol University, School of Pharmacy, Department of Pharmacognosy, Istanbul 34815, Turkey

[2]Molecular Targets Program, CCR, NCI, Frederick, MD 21702, USA

[3]Istanbul University, Faculty of Pharmacy, Department of Pharmacognosy, Istanbul 34116, Turkey

Correspondence

Prof. Dr. Mahmut Miski, Istanbul University, Department of Pharmacognosy, Istanbul 34116, Turkey

mahmud.miski@istanbul.edu.tr; Phone: +90-545-550-4455 Fax: +90-212-440-0252

Table of Contents

Fig. S1 Structures of sesquiterpene coumarins isolated from the root extract of *Heptaptera anatolica*........3

Fig. S2 ^{1}H NMR spectrum (600 MHz, $CDCl_3$) of anatolicin (**8**)........4

Fig. S3 ^{13}C NMR spectrum (125 MHz, $CDCl_3$) of anatolicin (**8**)........5

Fig. S4 2D COSY spectrum of anatolicin (**8**)........6

Fig. S5 2D HSQC spectrum of anatolicin (**8**)........7

Fig. S6 2D HMBC spectrum of anatolicin (**8**)........8

Fig. S7 2D NOESY spectrum of anatolicin (**8**)........9

Fig. S8 HRESIMS spectrum of anatolicin (**8**)........10

Fig. S9 ^{1}H NMR spectrum of Umbelliprenin (**1**)........11

Fig. S10 ^{1}H NMR spectrum of Karatavicinol (**2**)........12

Fig. S11 ^{1}H NMR spectrum of Badrakemone (**3**)........13

Fig. S12 ^{1}H NMR spectrum of Badrakemin (**4**)........14

Fig. S13 ^{1}H NMR spectrum of Colladonin (**5**)........15

Fig. S14 ^{1}H NMR spectrum of 14'-Hydroxycolladonin (**6**)........16

Fig. S15 ^{1}H NMR spectrum of 14'-Acetoxybadrakemin (**7**)........17

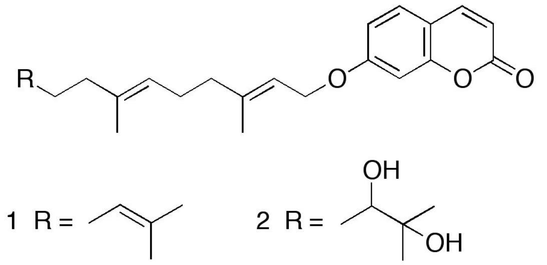

1 R =　　2 R =

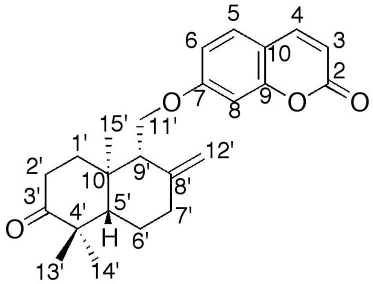

3

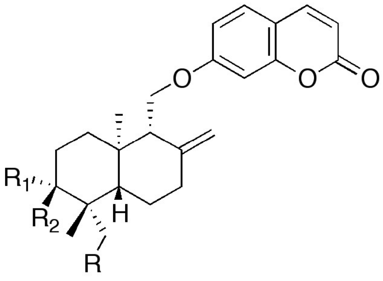

4　R = R_1 = H, R_2 = OH
5　R = R_2 = H, R_1 = OH
6　R = OH, R_1 = OH, R_2 = H
7　R = OAc, R_1 = H, R_2 = OH

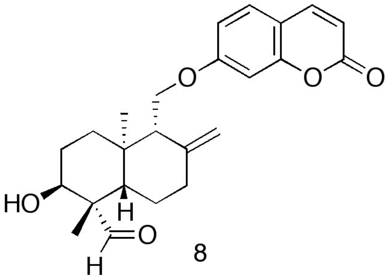

8

Fig. S1 Structures of sesquiterpene coumarins isolated from the root extract of *Heptaptera anatolica*.

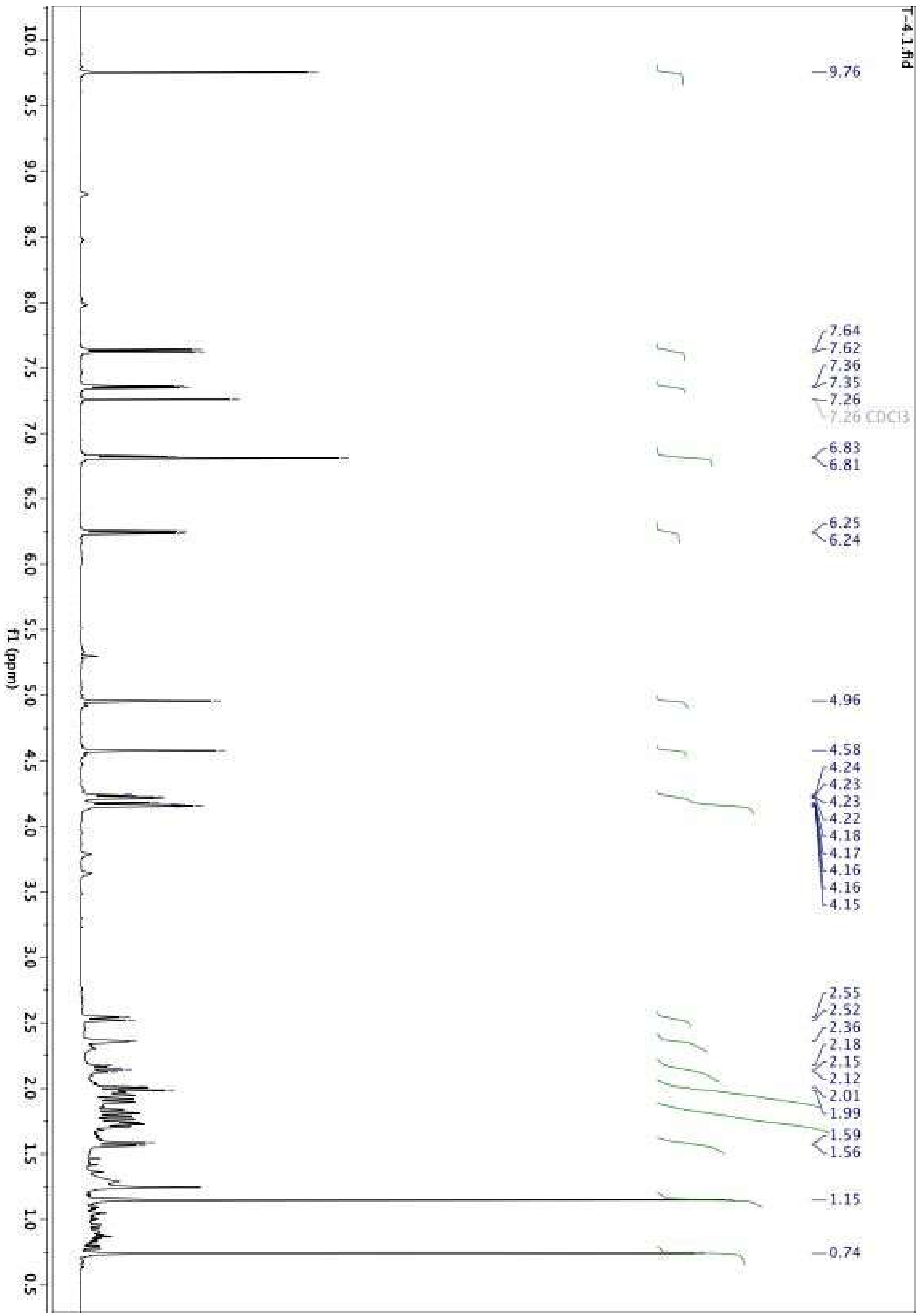

Fig. S2 ^{1}H NMR spectrum (600 MHz, $CDCl_3$) of anatolicin (**8**)

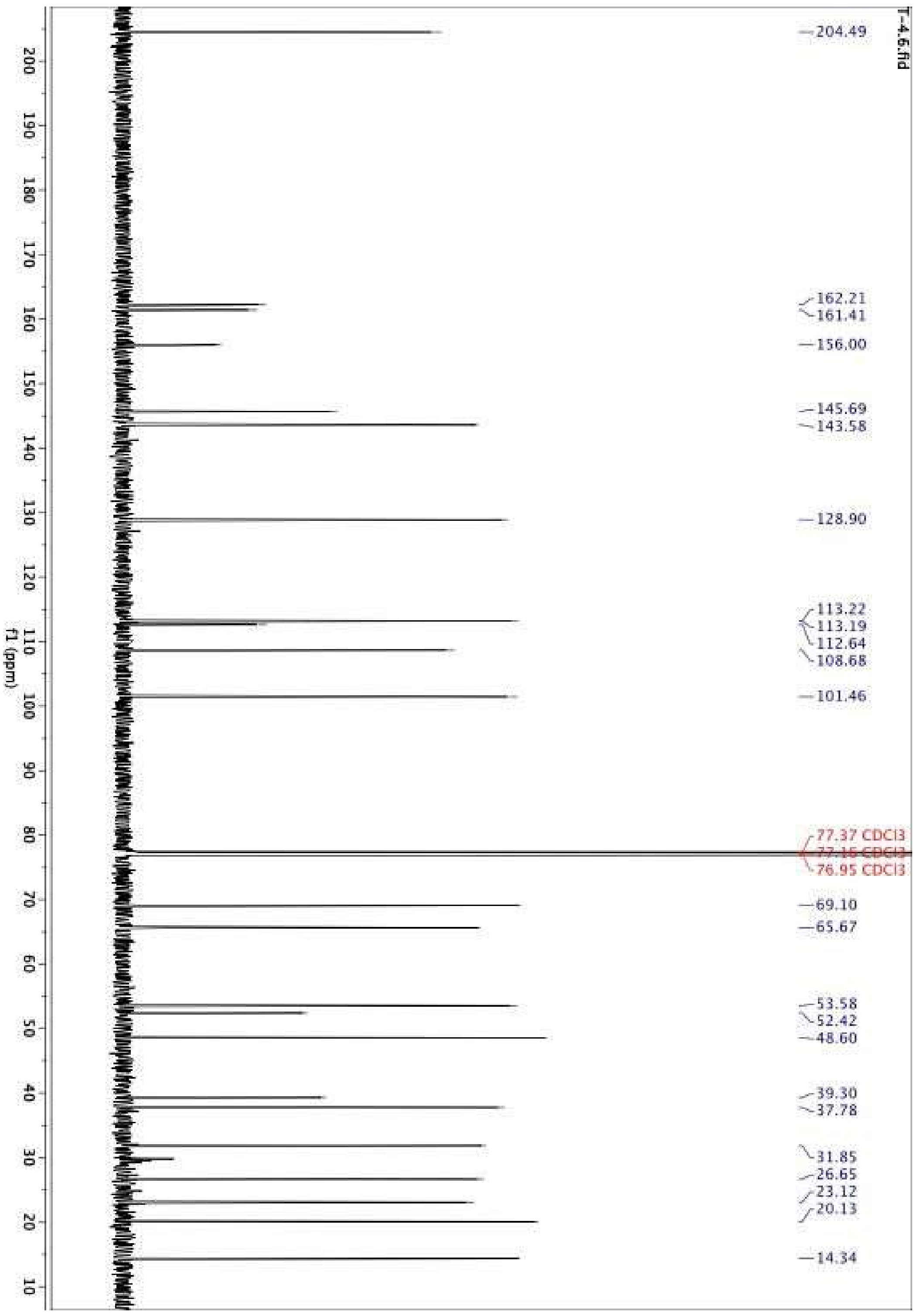

Fig. S3 ^{13}C NMR spectrum (125 MHz, $CDCl_3$) of anatolicin (**8**)

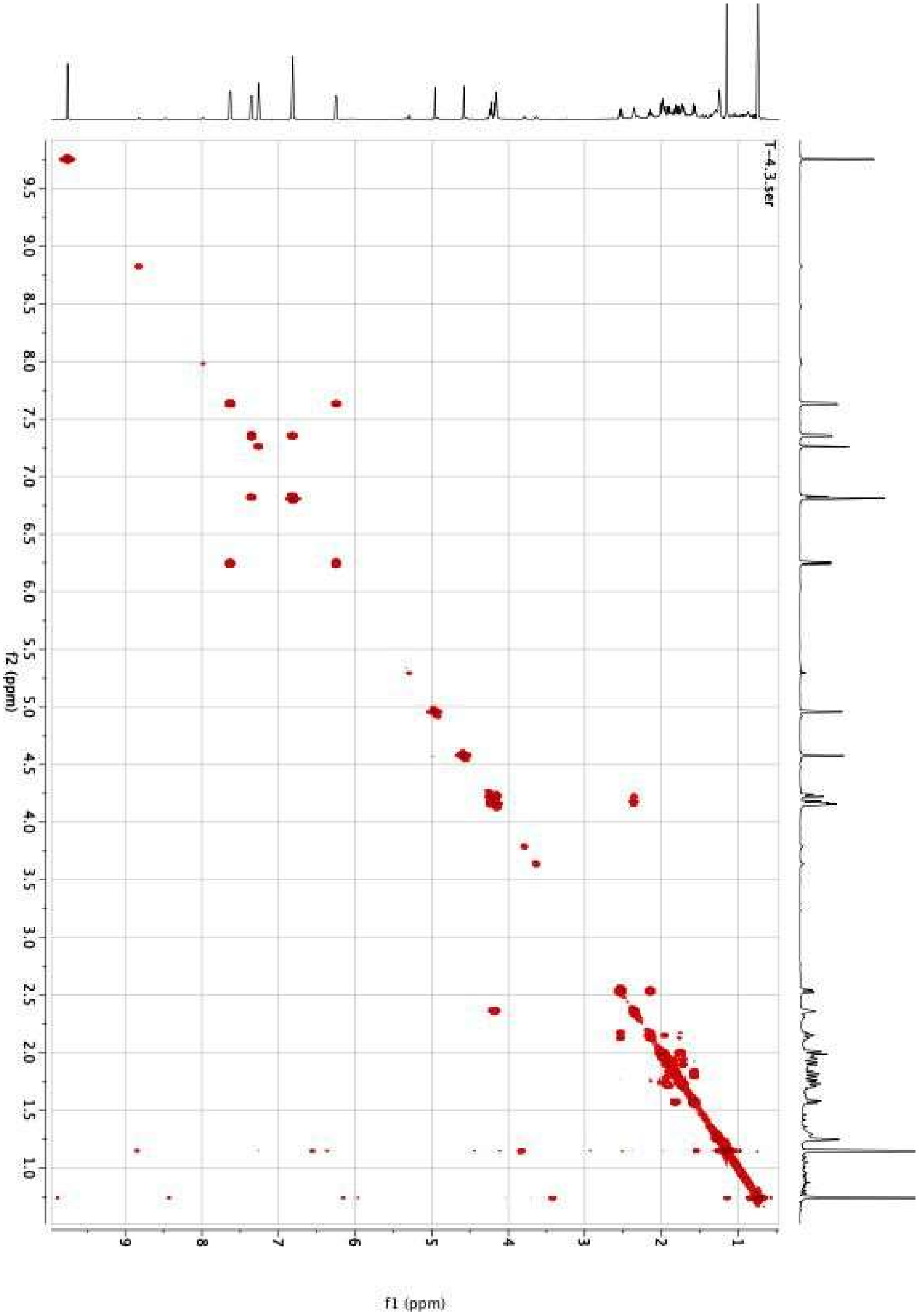

Fig. S4 2D COSY spectrum of anatolicin (**8**)

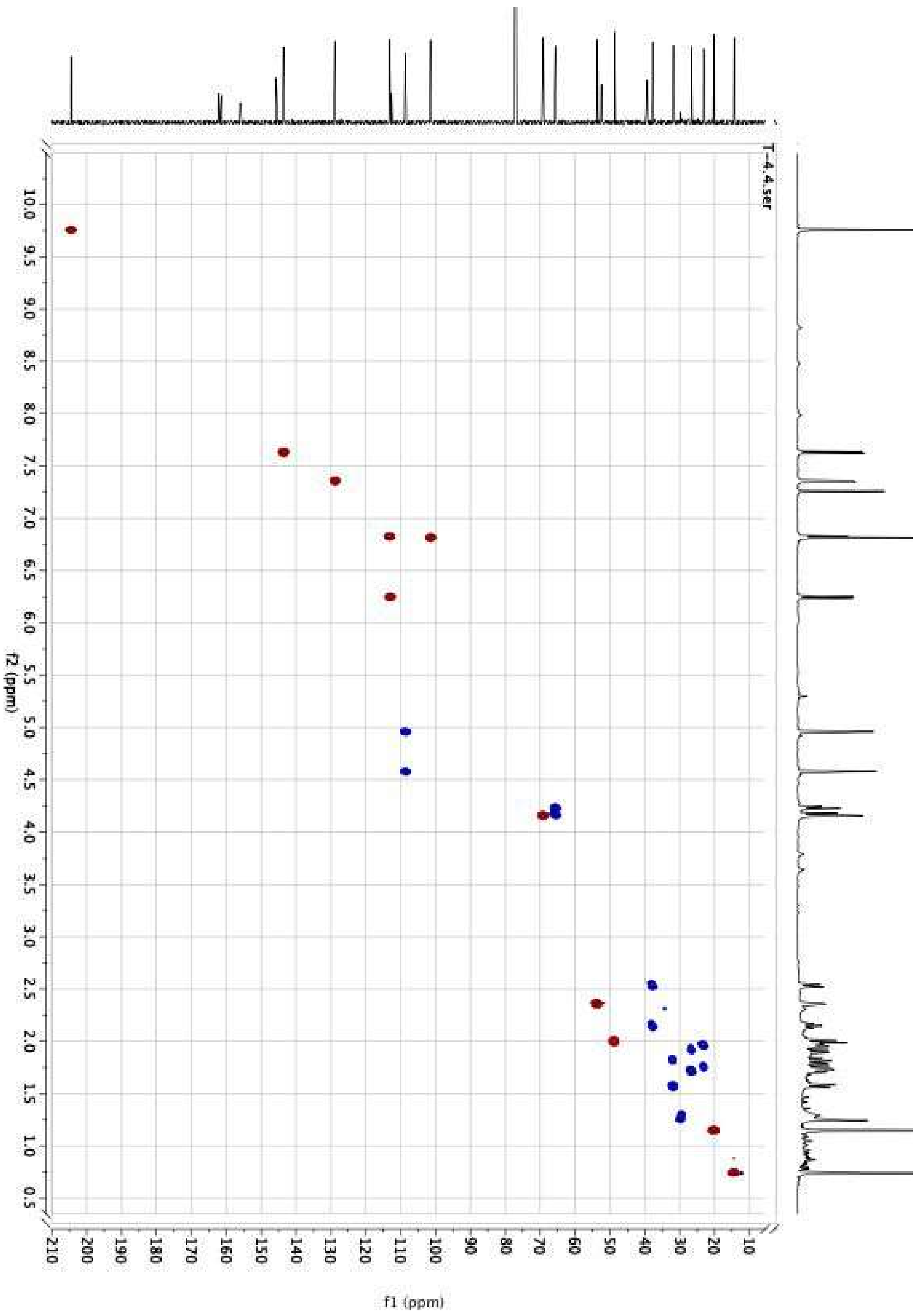

Fig. S5 2D HSQC spectrum of anatolicin (**8**)

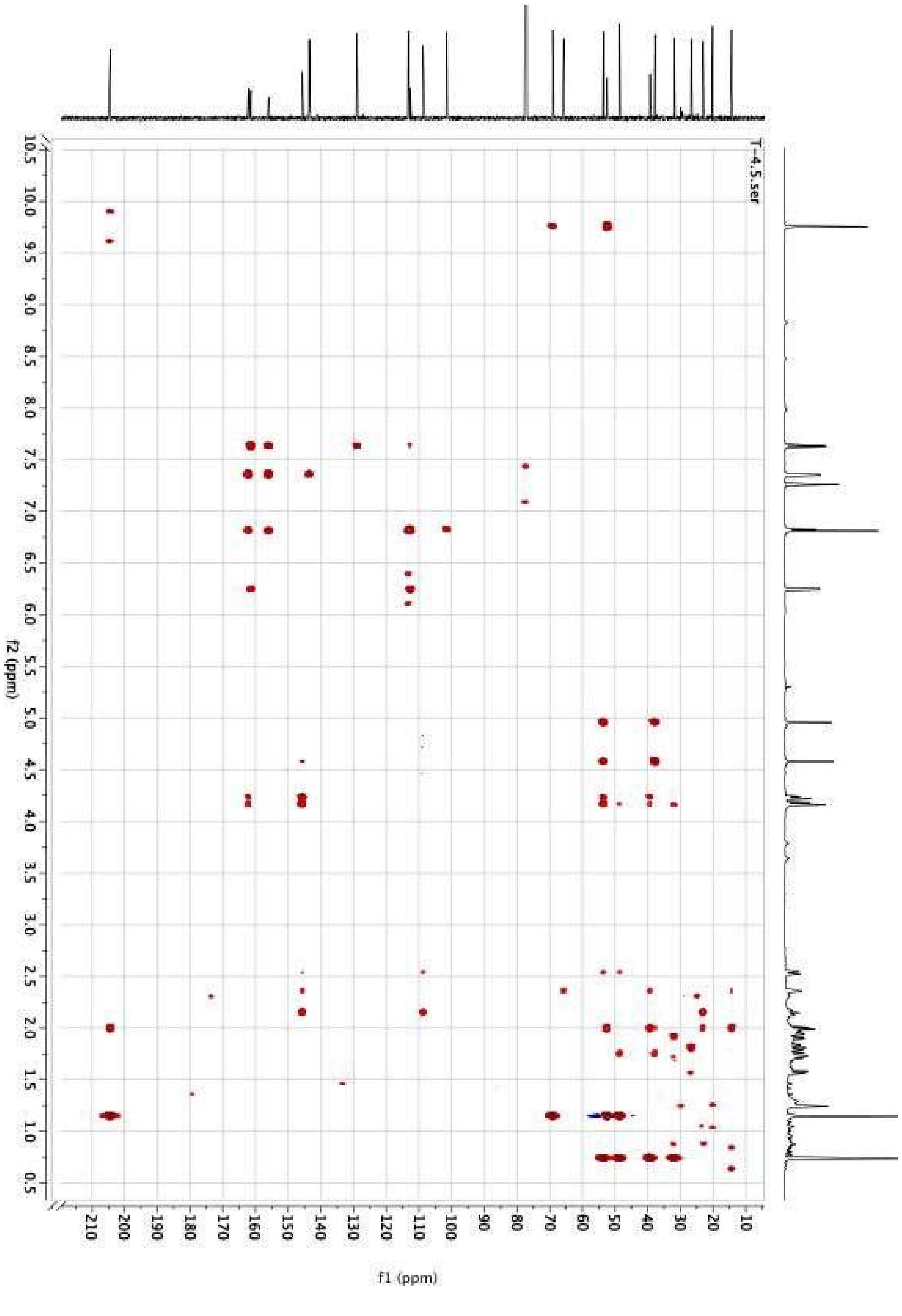

Fig. S6 2D HMBC spectrum of anatolicin (**8**)

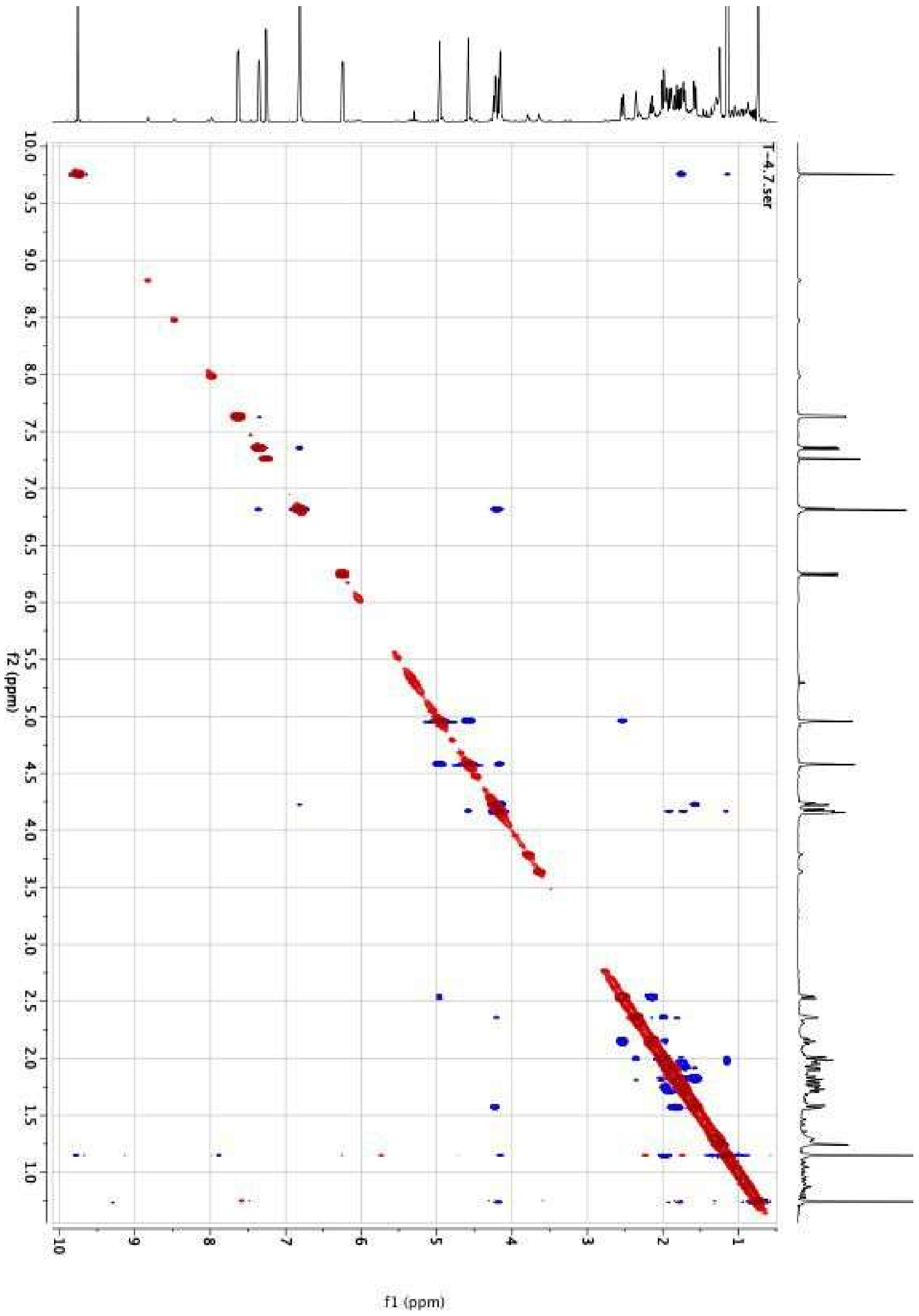

Fig. S7 2D NOESY spectrum of anatolicin (**8**)

Qualitative Compound Report

**Data File**	101I069F.d	**Sample Name**	101I069F
**Sample Type**	Sample	**Position**	Vial 41
**Instrument Name**	Instrument 1	**User Name**	Heidi
**Acq Method**	SMaccms_DESI(+)_Centroid.m	**Acquired Time**	1/15/2018 11:21:35 AM
**IRM Calibration Status**	Success	**DA Method**	Heidi.m
**Comment**			

**Sample Group**		**Info.**	
**Stream Name**	LC 1	**Acquisition SW Version**	6200 series TOF/6500 series Q-TOF B.06.01 (B6172 SP1)

Compound Table

Compound Label	RT	Mass	Formula	MFG Formula	MFG Diff (ppm)	DB Formula
Cpd 1: C24 H28 O5; 397.20095 396.19365	5.81	396.19365	C24 H28 O5	C24 H28 O5	0.05	C24 H28 O5

Compound Label	*m/z*	RT	Algorithm	Mass
Cpd 1: C24 H28 O5; 397.20095 396.19365	397.20095	5.81	Find by Molecular Feature	396.19365

MFE MS Spectrum

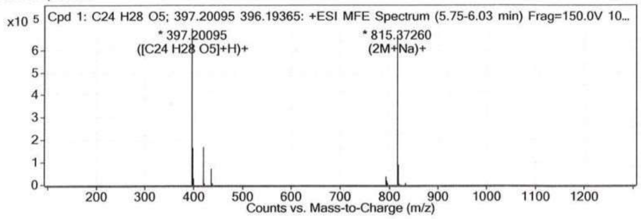

MFE MS Zoomed Spectrum

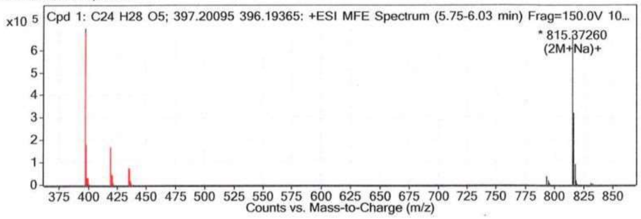

MS Spectrum Peak List

m/z	z	Abund	Formula	Ion
397.20095	1	695393.69	C24 H28 O5	(M+H)+
398.20425	1	164220.08	C24 H28 O5	(M+H)+
399.20696	1	26898.86	C24 H28 O5	(M+H)+
419.18266	1	169149.11	C24 H28 O5	(M+Na)+
420.18617	1	41114.52	C24 H28 O5	(M+Na)+
435.15636	1	72662.35	C24 H28 O5	(M+K)+

Agilent Technologies   Page 1 of 2   Printed at: 12:23 PM on:1/15/2018

Fig. S8 HRESIMS spectrum of anatolicin (**8**)

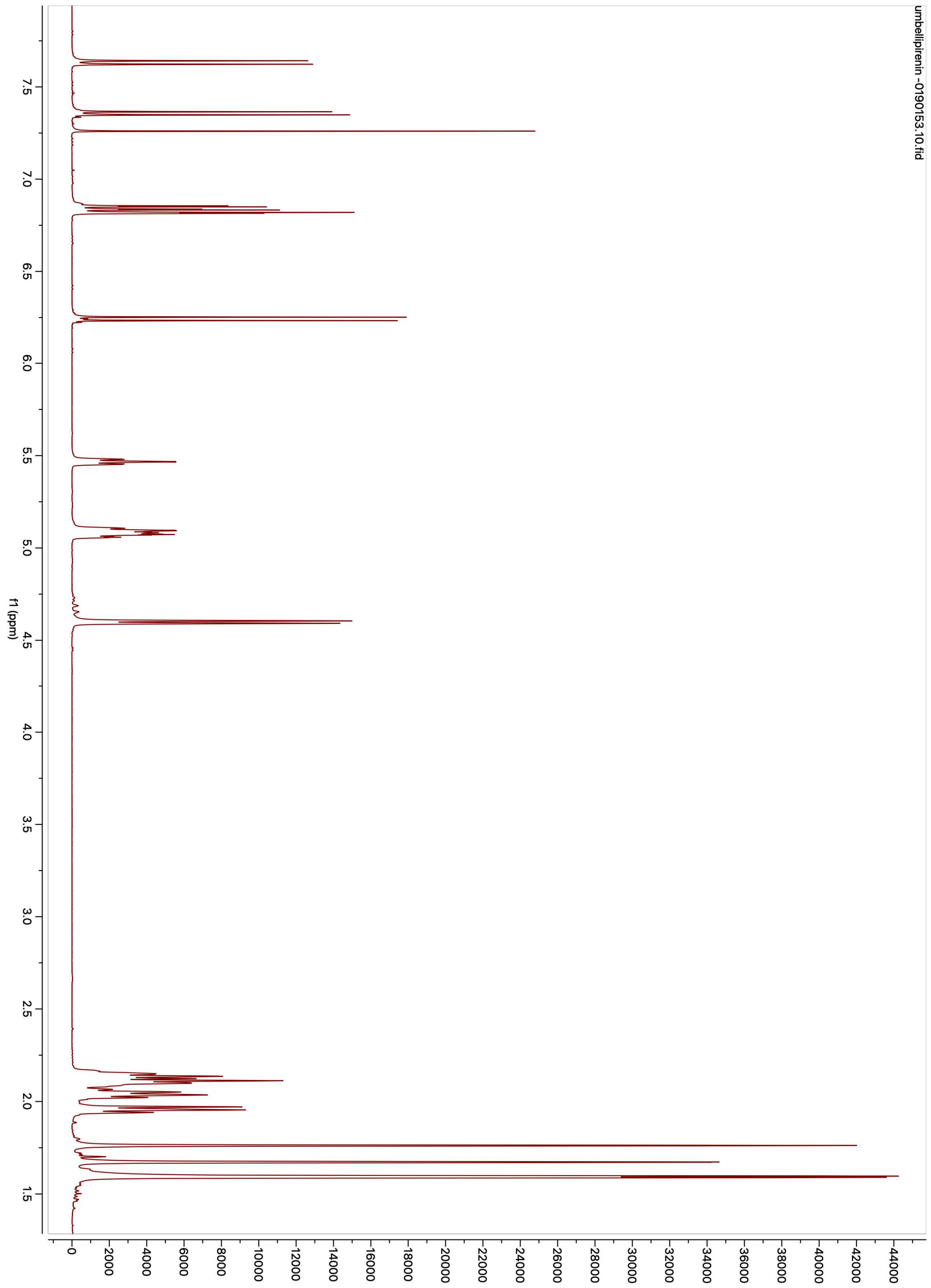

Fig. S9 ^{1}H NMR spectrum of Umbelliprenin (**1**)

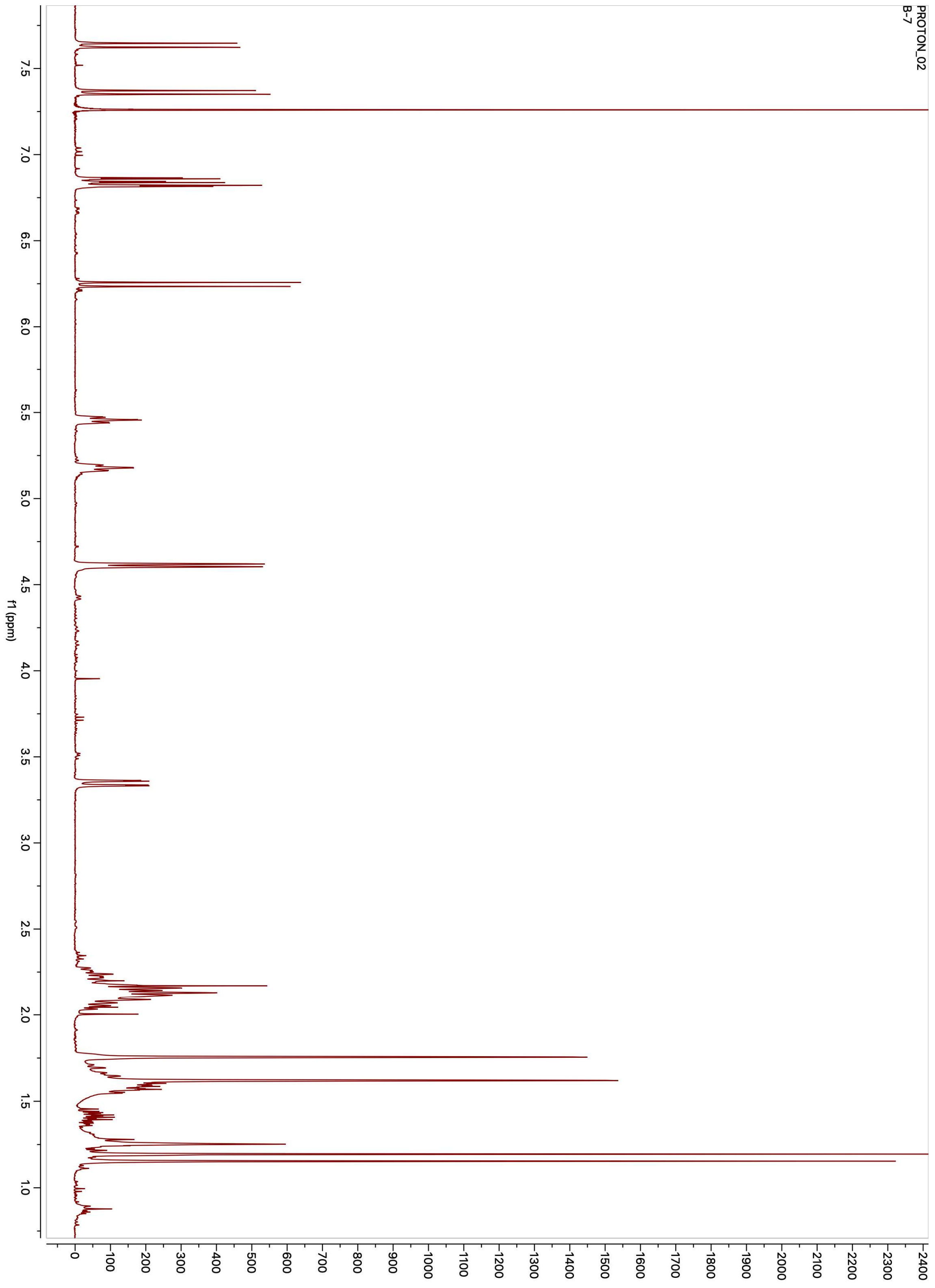

Fig. S10 ^{1}H NMR spectrum of Karatavicinol (**2**)

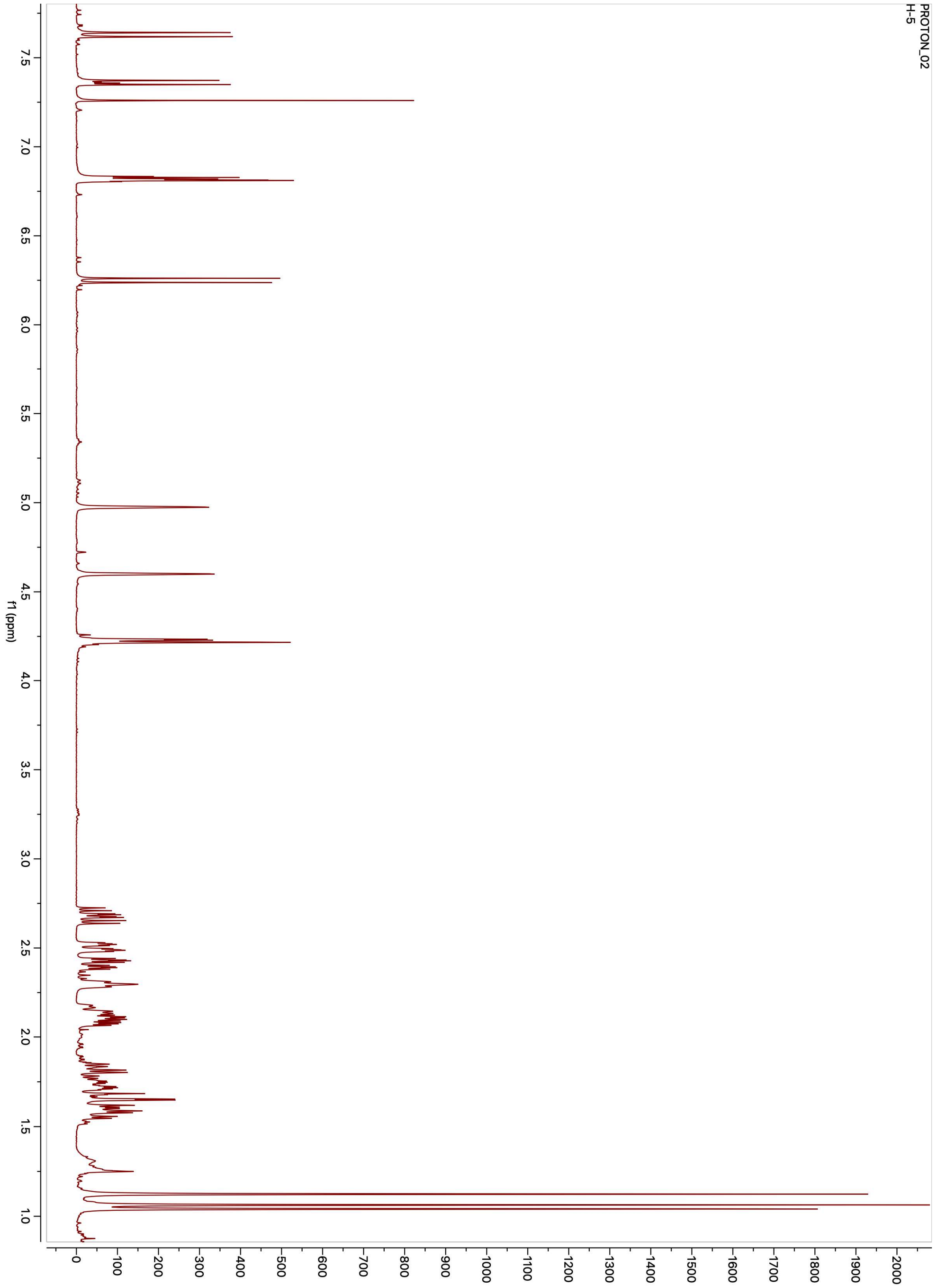

Fig. S11 ^{1}H NMR spectrum of Badrakemone (**3**)

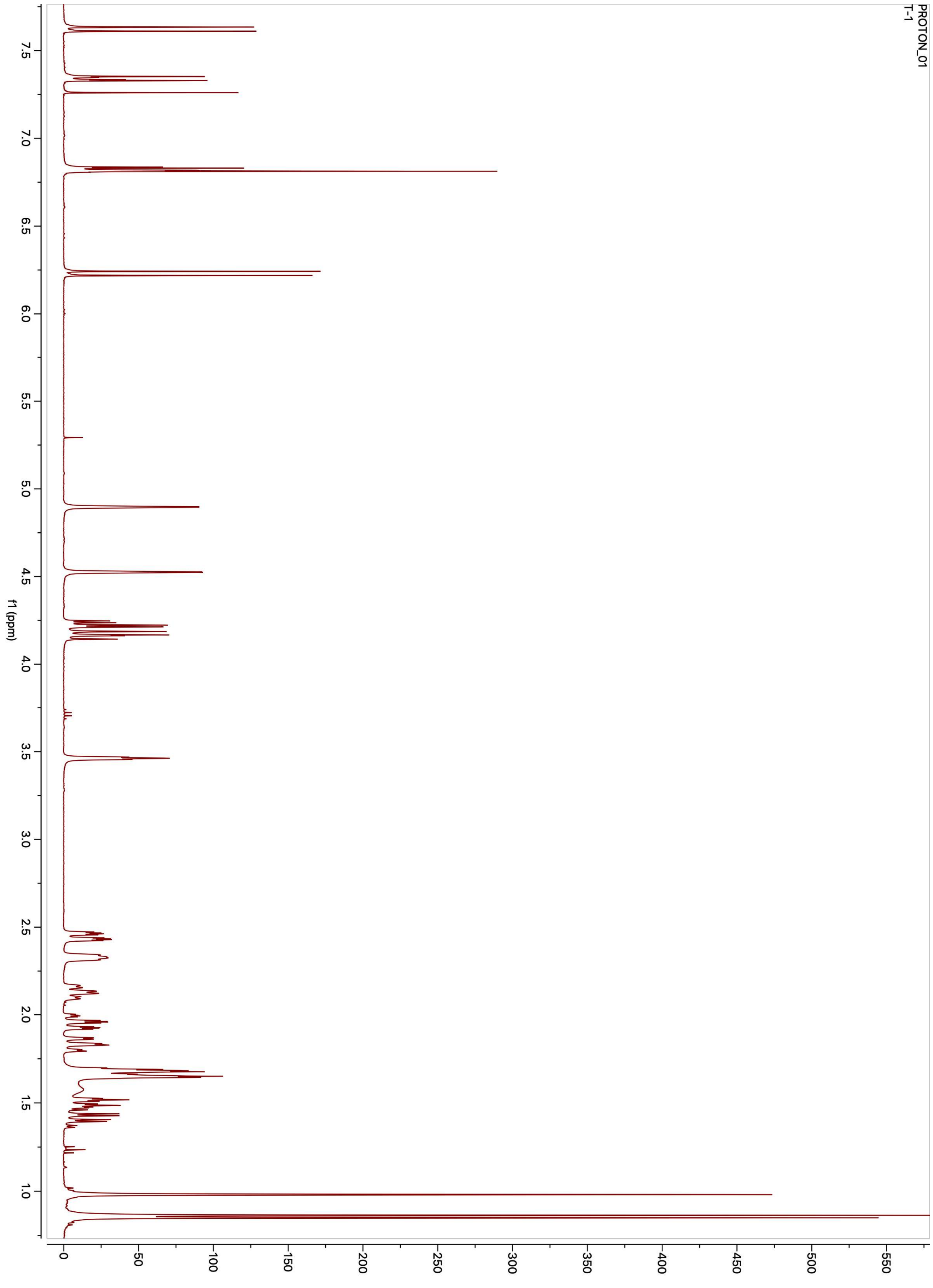

Fig. S12 ^{1}H NMR spectrum of Badrakemin (**4**)

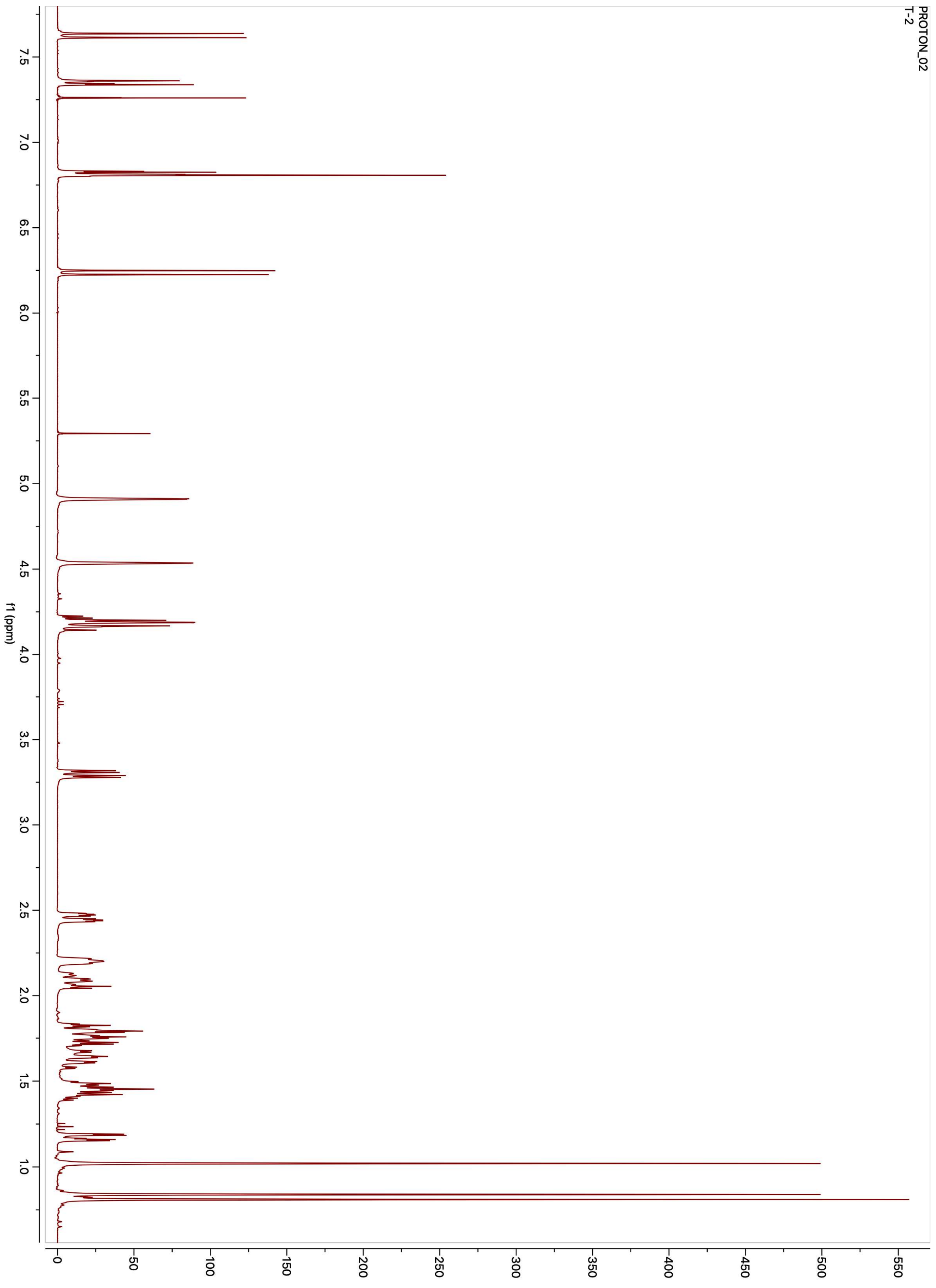

Fig. S13 ^{1}H NMR spectrum of Colladonin (**5**)

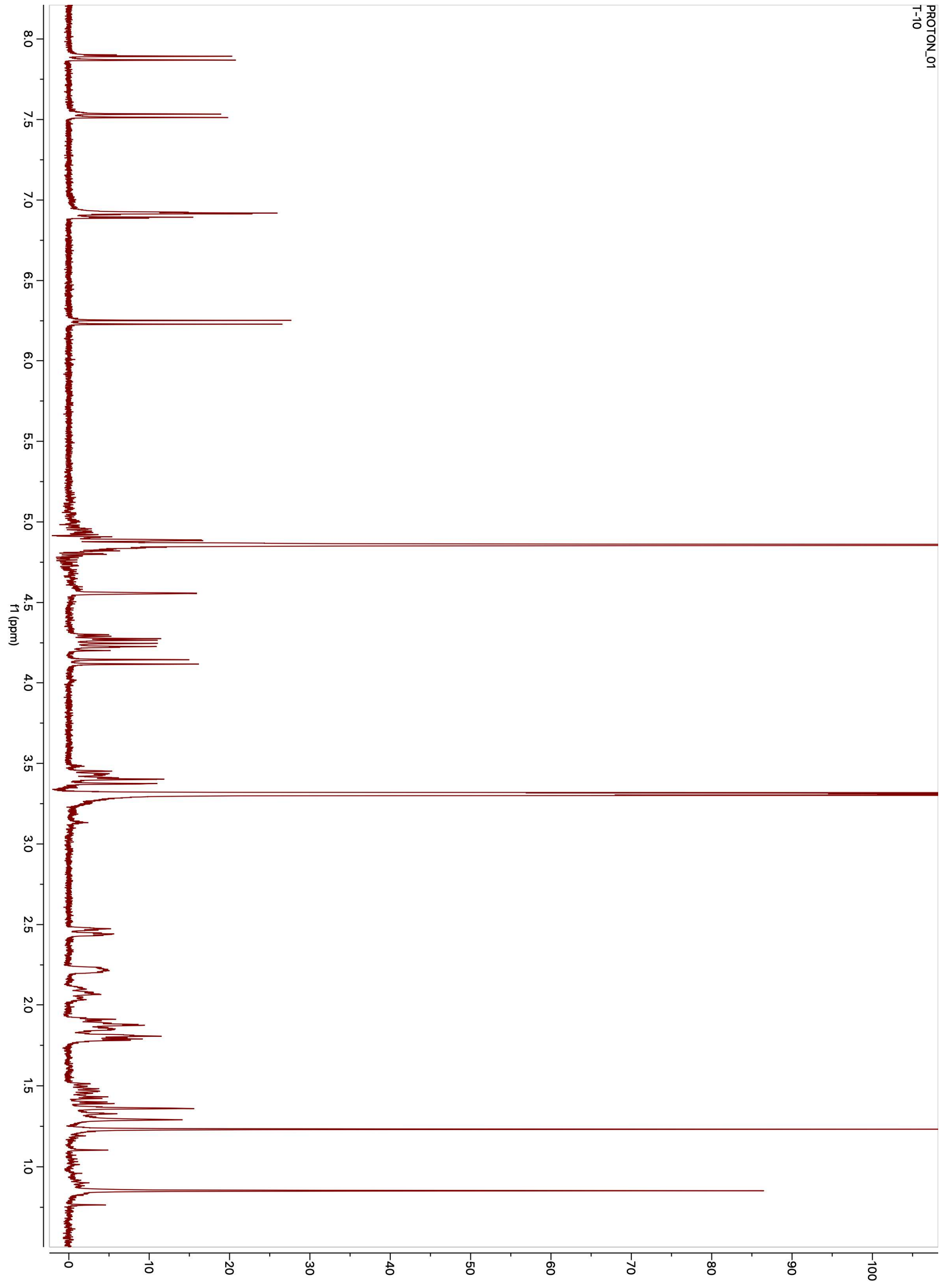

Fig. S14 ^{1}H NMR spectrum of 14’-Hydroxycolladonin (**6**)

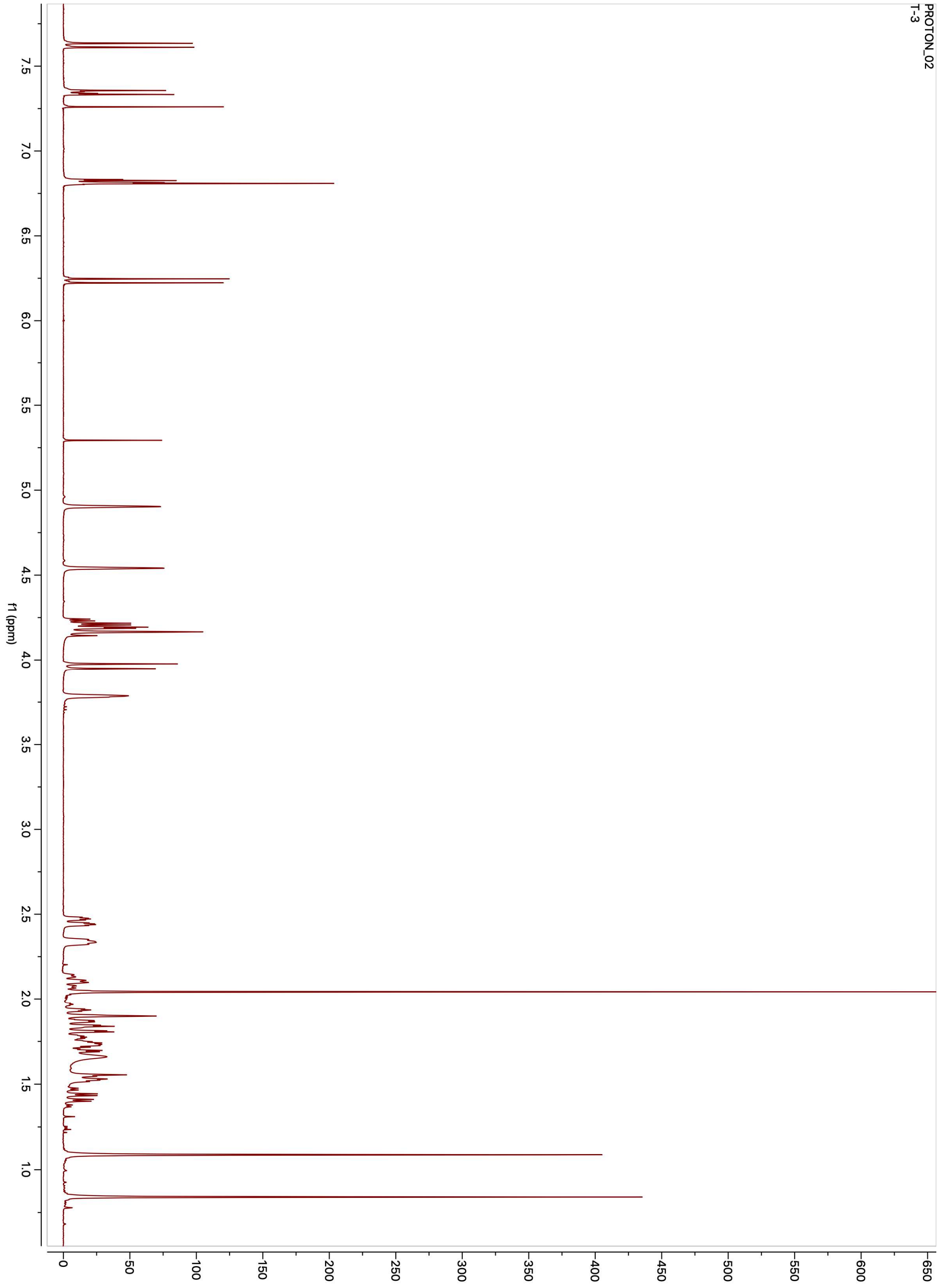

Fig. S15 ^{1}H NMR spectrum of 14’-Acetoxybadrakemin (**7**)
